# Promotion of peripheral nerve regeneration and prevention of neuroma formation by PRGD/PDLLA/β-TCP conduit: report of two cases

**DOI:** 10.1093/rb/rbv006

**Published:** 2015-06-01

**Authors:** Yixia Yin, Binbin Li, Qiongjiao Yan, Honglian Dai, Xinyu Wang, Jifeng Huang, Shipu Li

**Affiliations:** ^1^State Key Laboratory of Advanced Technology for Materials Synthesis and Processing, Wuhan University of Technology, Wuhan, 430070, China and ^2^Department of Orthopedic Surgery, Wuhan General Hospital of Guangzhou Military Command, Wuhan, 430070, China

**Keywords:** clinical case, peripheral nerve regeneration, neuroma formation, PRGD/PDLLA/β-TCP conduit

## Abstract

In the field of nerve repair, one major challenge is the formation of neuroma. However, reports on both the promotion of nerve regeneration and prevention of traumatic neuroma in the clinical settings are rare in the field of nerve repair. One of the reasons could be the insufficiency in the follow-up system. We have conducted 33 cases of nerve repair using PRGD/PDLLA/β-TCP conduit without any sign of adverse reaction, especially no neuroma formation. Among them, we have selected two cases as representatives to report in this article. The first case was a patient with an upper limb nerve wound was bridged by PRGD/PDLLA/β-TCP conduit and a plate fixation was given. After nearly 3-years’ follow-up, the examination results demonstrated that nerve regeneration effect was very good. When the reoperation was performed to remove the steel plate we observed a uniform structure of the regenerated nerve without the formation of neuroma, and to our delight, the implanted conduit was completely degraded 23 months after the implantation. The second case had an obsolete nerve injury with neuroma formation. After removal of the neuroma, the nerve was bridged by PRGD/PDLLA/β-TCP conduit. Follow-up examinations showed that the structure and functional recovery were improved gradually in the 10-month follow-up; no end-enlargement and any other abnormal reaction associated with the characteristic of neuroma were found. Based on our 33-case studies, we have concluded that PRGD/PDLLA/β-TCP nerve conduit could both promote nerve regeneration and prevent neuroma formation; therefore, it is a good alternative for peripheral nerve repair.

## Introduction

The reconstruction of injured peripheral nerve and the prevention of neuroma formation after surgery are still the big challenges in the field of nerve repair [1–6]. Although the autografting has been the golden standard for the nerve reconstructive strategy, the therapeutic effects remain problematic due to the limitations from the donors and the formation of neuroma after the injury [7, 8]. In particular, neuroma formation could potentially cause the patient loss of sensory, neuropathic pain or the need for additional surgery [9]. Neuroma generally forms after connective tissue hyperplasia caused by the inflammatory reaction or high concentration of nerve growth factor (NGF) [10, 11]. Therefore, finding an alternative that can both promote nerve regeneration and prevent neuroma growth has become an urgent mission for the researchers. Various nerve conduits made by natural materials or artificial composites have been developed and tested in bridging peripheral nerve defects in many animal models [12–15]. The length of the conduits ranged from 10 to 80 mm, some have achieved similar outcomes as autografting [16–18]. In addition, silicon and bioabsorbable conduits have been used in bridging the large-diameter nerve defects of digital, median and ulnar nerves at wrist or distal forearm in the clinical settings [19].

Previous studies have shown encouraging results and provided pioneered examples for the tubular repair of peripheral nerve injuries, especially in the cases related to the issue of the neuroma formation after the repair [1, 9, 19]. We have developed a new type of artificial nerve graft comprising of PRGD/PDLLA/β-TCP conduits. Poly (*d,l*-lactic acid) (PDLLA) showed good biocompatibility with nerve tissues and cells, and it is biodegradable [20]. The β-tricalcium phosphate (β-TCP) can improve the cell attachment and balance out the acid degradation product released from PDLLA [21]. The (lactic acid)-co-[(glycolic acid)-alt-(L-lysine)] (RGD) motif has been not only proven to enhance the attachment and elongation of Schwann cell *in vitro* [22, 13], but also capable of facilitating the axon growth in the early stages across rat sciatic transaction injuries *in vivo* [23, 24]. In our previous report, after the implantation of PRGD/PDLLA/β-TCP conduit, the interluminal NGF concentration of conduit was decreased over time, especially after 7 days. Compared with PDLLA only conduit, our earlier research [25-27, 21] also showed that PRGD/PDLLA/β-TCP conduit has better biocompatibility and less inflammatory reaction, both of which are important factors in the prevention of neuroma formation.

The PRGD/PDLLA/β-TCP conduit have been used to bridge implantation of rat [21] and dog sciatic nerves, and the longest defect was 30 mm in length across the single largest nerve trunk in a dog [28]. In addition, a clinical trial was set up to examine the feasibility of PRGD/PDLLA/β-TCP conduit in major peripheral nerves repair. We have so far performed repair surgeries in 33 cases without any adverse reaction.

In this article, two cases representing different types of nerve damage were presented out of total of 33 cases underwent the surgical repairs with PRGD/PDLLA/β-TCP artificial nerve grafting. One case involved a patient with a 25-mm-long nerve defect in the radial nerve, and the other patient had a 10-mm defect in median nerve with the growth of the neuroma. To evaluate the post-surgery nerve regeneration and functional recovery, we have designed a 3-year follow-up plan using the standard diagnostic tools, such as electrophysiology, high frequency B ultrasonography, clinical sense and movement assays, and etc. Our results strongly suggested that the functional recovery of the repaired nerve was very successful in patients using PRGD/PDLLA/β-TCP conduit. 

The ethics committee in the General Hospital of Guangzhou military command has reviewed and approved these cases for publication.

## Clinical Materials and Methods

This clinical research was conducted in the Wuhan General Hospital of Guangzhou military command and Pu’ai Hospital. There were total of 33 clinical cases with inductive repairs of peripheral nerve: 16 cases had fresh injury; and 17 cases suffered from obsolete nerve defects, of them, 13 cases had the formation of the neuroma. The repaired nerves were all ≤2.5 cm in length with large diameters. Follow-up examinations were scheduled regularly for 3 years. Routine functional recovery examination and evaluation included electrophysiology, high frequency B ultrasonography and clinical sensational exercise tests.

The clinical evaluation was conducted according to the standard of Chinese medical association for upper part of neural function, and the motor grading system recommended by the British Medical Research Council. Each patient was given ratings or scores based on the level of functional recovery, including the sensory, movement and overall comprehensive evaluation, etc.

## Case Reports

### Patient 1: radial nerve injury

**Figure 1:**
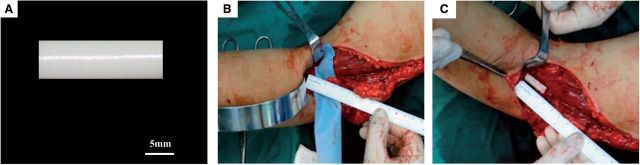
Gross image of PRGD/PDLLA/β-TCP conduit and photographs taken during the operation. (**A**) Gross image of PRGD/PDLLA/β-TCP conduit; (**B**) 20-mm-long nerve defect was bridged with PRGD/PDLLA/β-TCP nerve conduit (25 mm in length and 5 mm inner diameter) (**C**).

**Figure 2:**
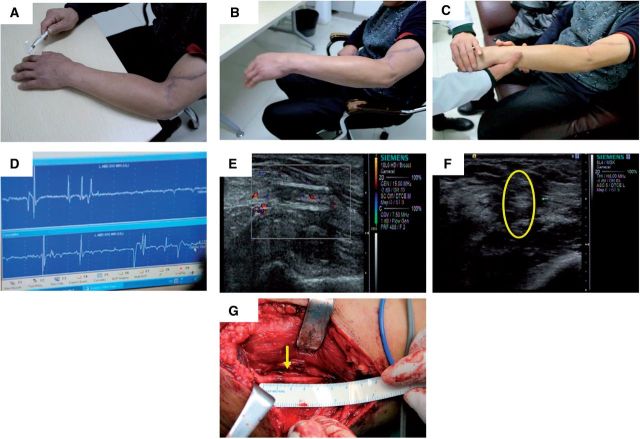
Follow-up examination of patient 1. (**A**) Monofilament touch test was conducted 13 months after the operation. The measurement was 300 g in tiger mouth area, and 0.4 g in proximal phalanx of index and middle finger; (**B**) Photographs showing the appearance and functional recovery 13 months after the operation. Wrist stretch and finger stretch of left hand has almost returned to the normal level. Myodynamia reached level 4, and (**C**) the patient could stretch wrist and fingers in the presence of strong resistance; (**D**) Electrophysiology results 13 months after the operation. As measured by EMG, the motor nerve conduction velocity was 65.8 m/s, which was close to the normal level; (**E, F**) High Frequency B Ultrasonography 13-months after the operation. The defects have been bridged with the regenerated nerve. The regenerated nerve fibers appeared healthy with whole lumen and a clear appearance. The blood flow to the nerve was abundant, and no swelling was observed in the conjunction area; (**G**) Photograph taken during the surgery to remove the Jefferson-fracture reduction plate 23 months after the implantation. The nerve was completely regenerated and reconnected to the both ends of the defects; the structure appeared uniformly without any abnormal expansion. The conduit material was not visible, presumably has degraded completely.

The patient was a 49-year-old man suffered from a chop knife wound resulting in the breakage of radial nerve at his left elbow. Wrist drop of left hand was observed, wrist and finger could barely stretch. In addition, the thumb-index, proximal phalanx of index finger and middle finger lacked sensation. The operation to repair the radial nerve was conducted on 10 December 2011. A 20-mm-long defect was measured in the radial nerve (Fig. 1B) and were bridged with PRGD/PDLLA/β-TCP nerve conduit (5 mm inner diameter and 25 mm length), shown in Fig. 1C. Neuromuscular function was regained gradually after the operation. Follow-up examinations were done 3 months after the operation. Tinel test on the injured nerve site of left elbow was positive, and conduction signal from the nerve can be electrophysiological detected. The patient reported numbness that expanded to his thumb-index area. At the 6 months follow-up, sensory nerve conduction velocity returned to near normal level at 48 m/s as examined by the electrophysiology tests, and the functions of both wrist and finger stretch have partially recovered. High frequency ultrasonography showed that the regeneration of nerve fiber growing along conduit wall from near to far end. The blood flow was detected at the far-end of the nerve as well. The results from the follow-up 7.5 months after the operation showed that the ability to stretch wrist and finger was drastically improved. High frequency B ultrasonography confirmed the regeneration of nerve fiber growing from the tube wall of PRGD/PDLLA/β-TCP to the lumen center with abundant blood flowing in. The next follow-up was 13 months after the operation. The measurement from the monofilament touch testing was 300 g in tiger mouth area and 0.4 g in proximal phalanx of index and middle finger (Fig. 2A). Two-point sensory discrimination test was used to check the recovery of the sensory nerve function, and the readout was 10 mm for the thumb-index area, 6 mm for the proximal phalanx of index finger and 4 mm for proximal phalanx of middle finger. The stretches of the wrist and finger has returned to the normal level. Myodynamia has reached to level 4, and the patient could stretch his wrist and fingers even in the presence of strong resistance (Fig. 2B and C). Electromyography (EMG) procedure also showed that the motor nerve conduction velocity reached 65.8 m/s, very close to the normal level (Fig. 2D). High frequency B ultrasonography again reinforced the fact that the regenerated nerve tracts between the near-end and far-end have been bridged (Fig. 2E), regenerated nerve fibers were full of whole lumen, the regenerated nerve in conduit was clear, and there were sufficient blood flowing into the nerve. The overall nerve regeneration was good without any swelling in the broken ends or around the regenerated nerve (Fig. 2F). At 23 months after the operation, the stretch functions of the wrist and finger has fully recovered that they could move freely. Internal and external rotation of forearm returned to normal. When the fracture fixation steel plate was surgically removed, we have observed that nerve regeneration was very good with no signs of neuroma. Moreover, PRGD/PDLLA/β-TCP nerve conduit has been completely degraded and absorbed (Fig. 2G). Pathological analysis on the surrounding tissues did not show any untoward effect.

### Scores at 23 months after the operation

Wrist stretch was >45° (4 points); myodynamia was greater than M3 (4 points); The total active motion (TAM) activity for thumb stretch returned to the normal range (excellent, 4 points); TAM activity for the finger stretch scored >75% of uninjured side (good, 3 points). The combined total score was 15 with an excellent rating.

### Patient 2: median nerve injury

**Figure 3:**
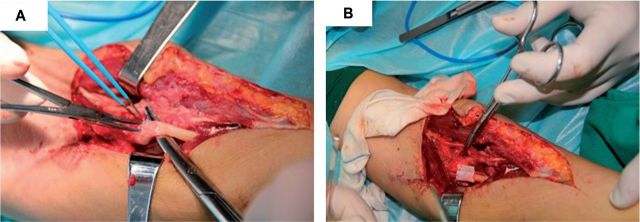
Photographs taken during the operation. (**A**) Obsolete nerve injury with neuroma formation; (**B**) Nerve was bridged by PRGD/PDLLA/β-TCP conduit after removal of the neuroma.

A 54-year-old woman sustained a wound on the middle and upper section of left forearm with median nerve injury, and an operation for nerve detection repair were initially performed at another hospital. Eight months after the surgery, her left hand fingers still could not reach her palm, the external stretch was limited, and sensory in thumb, first and middle finger was lost. During the operation, nerve tumor was observed around the broken nerve ends (Fig. 3A). The neuroma was removed, resulting in a 1 cm defect in the median nerve. To bridge the two ends of nerves, PRGD/PDLLA/β-TCP nerve conduit (5 mm inner diameter and 15 mm length) was used (Fig. 3B).

**Figure 4:**
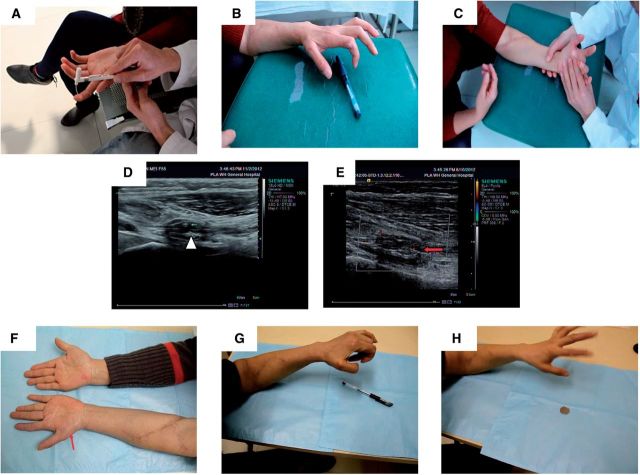
Follow-up examinations of patient 2. (**A**) Monofilament touch test 10 months after the operation. Photograph shown that monofilament touch pressure test was given to the patient; (**B, C**) Photographs showing the appearance and functional recovery 6 months post-operation. (**B**) Pen picking was demonstrated; (**C**) Wrist bending was done with little assistant; (**D, E**) High Frequency B Ultrasonography taken 6 months after the operation. (**D**) The triangle arrow pointed to the nerve fibers tract located in the conduit; (**E**) the nerve between the distal and proximal ends has been connected, red arrow indicated the blood flow in median nerve located at the distal end of the conduit; (**F**) The amyotrophy of thenar muscles at the left have greatly improved 10 months after the operation; (**G, H**) Functional Recovery at 10 months after the operation. (**G**) The left thumb can hold a pen firmly with another finger in fluent and coherent action. (**H**) Demonstrated that the finger can hold a coin (fine objects) with another finger with less smooth action.

Regular follow-up examinations were conducted postoperatively. First at 3 months after the operation, monofilament touch pressure test was conducted, and the measurements on distal phalanx of index and middle finger had a failed score, while the wrist joints position was 2.0 g, and palmar opposition of left thumb has partially recovered. The function of distal phalanx of index finger and middle finger improved greatly at 6 months post-operation, the measurement from monofilament touch pressure test scored at 300 g (Fig. 4A). However, the nerve conduction signals were still weak. Palmar opposition of left thumb has showed significant improvement, and the patient could hold a pen with her thumb and any other finger (Fig. 4B). Without any external assistant, wrist bending of left hand has recovered ∼80% as compared with that of the normal side (Fig. 4C).High frequency B ultrasonography confirmed the thick nerve fibers tract in the conduit (Fig. 4D), and broken nerves between the near and far ends have been connected. Regeneration of blood supply was also detected in the median nerve on far-end of the conduit (Fig. 4E). Follow-up examination at 10 months post-operation, the measurement for distal phalanx of index finger was 4.0 g and that for distal phalanx of middle finger was 300 g in the monofilament touch pressure test. Electrophysiological examination showed that motor nerve conduction velocity had recovered (29.80 m/s). The physical appearance of the left hand improved as well, and the amyotrophy of thenar muscles of left hand has recuperated compared with that at 6 months post-operation (Fig. 4F). Palmar opposition and flexion of left thumb was better than that in 6 months after the operation. The left thumb can not only hold a pen firmly with any other finger in fluent and coherent action (Fig. 4G), but also can hold a coin (fine objects), though the action remained unsmooth (Fig. 4H).

### Scores at 10 months after the operation

Superficial sensory of pain and touch in median nerve innervation area have recovered completely without any irritation (S3, 3 points). Myodynamia of the bent wrist was M4 (a score of 4), and the TAM activity of the bent finger returned to the normal range (excellent, a score of 4); Palmar opposition of the thumb was close to normal (a score of 4). Combined all the data earlier, the total score was 15 with excellent rating.

As the current golden standard for nerve regeneration restoration, autologous nerve graft has a recovery rate that is <70%. In the cases which PRGD/PDLLA/β-TCP composite conduit was used to repair nerve injury, the recovery rate was 78.9%, which is a better clinical repair outcome.

## Discussion

The median and ulnar nerves are the two major nerves in the upper extremities with a mixture of motor, sensory and sympathetic nerve fibers [29, 30]. Damage to those nerves could greatly impair a patient’s quality of life. To repair the peripheral nerve, successful nerve regeneration is the key to its functional recovery. This is especially important for the median nerve, which is responsible for most of the hand functions. The neuroma formation from old injured nerves represents a big challenge for the success of nerve regeneration. Due to current limitations, evaluation of the nerve regeneration could only be performed indirectly, relying on the functional data to monitor the recovery progress [10, 31]. Therefore, in this study, the commonly used diagnostic tools, such as high frequency B ultrasonography, grading system, sensation examinations and electrophysiology, were employed to investigate the functional recovery of the sensory and motor nerves.

In the first case, patient with radial nerve breakage had a sensory nerve conduction velocity of 48 m/s, which is close to the normal level; The motor nerve conduction velocity was also returned to normal at 65.8 m/s 13 months post-implantation of the conduit, and no side effect was found. The overall score was 15 and rated excellent at 23 months post-operation. Although performing the operation to remove the Jefferson-fracture reduction plate, it was observed that the regenerated nerve was connected between the two ends of the defects; the conduit material had degraded completely without any neuroma formation.

In the second case, the female patient with neuroma formation was treated, and the follow-up 6 months post-implantation showed that the function of active wrist had recovered to 80% compared with the normal side. The wrist-bend myodynamia has regained to M4 level, which is typical among the patients going through the restoration of the digital opposition and coordination of muscles. At 10 month post-implantation, the range of finger-bending TAM activity was normal (excellent, 4 points), and palmar opposition of thumb returned to the normal level (4 points). The total score was 15 with an excellent rating, indicating the overall functional restoration of motor and sensory nerves, as well as the gradual maturations of both the regenerated myelin and the regenerated axon. In addition, the regained glossy appearance on the radial part of the left palm suggested that the functions of sympathetic nerve have also been restored to a certain extent. No terminal expansion was observed and no discomfort was informed, suggesting that the area of repaired nerve was free of neuroma formation.

How well a patient can recover functionally after a nerve injury depends on many factors, such as the age of the patient, nerve type, denervation time, the length of the defect, injury level, and etc. [32]. The formation of neuroma is the major stumbling block in the recovery process after the surgery. Previous reports have shown that the nerve conduit can prevent neuroma formation and the ingrowth of fibrous tissue [33, 34]. PRGD/PDLLA/β-TCP conduit represents one of the latest classes of the artificial nerve grafts used in the peripheral nerve repair. The cases presented in this article have demonstrated that bridging of the injured nerves by this type of conduit resulting in good functional recovery without neuroma formation.

## Conclusion

The case studies we presented here strongly suggest that PRGD/PDLLA/β-TCP artificial nerve graft could be an effective nerve graft against poor nerve regeneration and against neuroma formation, making it a valid alternative for peripheral nerve repair. To better determine the clinical implications of this graft, more cases will be conducted and monitored. In addition, the follow-up period will be extended to monitor the long term outcomes.

## Funding

This work was supported by the National Basic Research Program of China (973 Program, No. 2011CB606205) and National Natural Science Foundation of China (No. 51403168, 81190133 and 51172172); Natural Science Foundation of Hubei Province (No. 2013CFB354) and Key grant Project of Chinese Ministry of Education (No. 313041).

*Conflict of interest statement*. None declared.
